# Self-powered wearable biosensor based on stencil-printed carbon nanotube electrodes for ethanol detection in sweat

**DOI:** 10.1007/s00216-024-05467-7

**Published:** 2024-08-12

**Authors:** Verdiana Marchianò, Angelo Tricase, Eleonora Macchia, Paolo Bollella, Luisa Torsi

**Affiliations:** 1 Department of Pharmacy-Pharmaceutical Science, University of Bari Aldo Moro, Via E. Orabona, 4, 70125 Bari, Italy; 2 Centre for Colloid and Surface Science, University of Bari Aldo Moro, Via E. Orabona, 4, 70125 Bari, Italy; 3 Faculty of Science and Engineering, Åbo Akademi University, 20500 Turku, Finland; 4 Department of Chemistry, University of Bari Aldo Moro, Via E. Orabona, 4, 70125 Bari, Italy

**Keywords:** Wearable biosensors, Water-based conductive inks, Modified electrodes, Ethanol biosensors, Enzymatic biofuel cells

## Abstract

Herein we introduce a novel water-based graphite ink modified with multiwalled carbon nanotubes, designed for the development of the first wearable self-powered biosensor enabling alcohol abuse detection through sweat analysis. The stencil-printed graphite (SPG) electrodes, printed onto a flexible substrate, were modified by casting multiwalled carbon nanotubes (MWCNTs), electrodepositing polymethylene blue (pMB) at the anode to serve as a catalyst for nicotinamide adenine dinucleotide (NADH) oxidation, and hemin at the cathode as a selective catalyst for H_2_O_2_ reduction. Notably, alcohol dehydrogenase (ADH) was additionally physisorbed onto the anodic electrode, and alcohol oxidase (AOx) onto the cathodic electrode. The self-powered biosensor was assembled using the ADH/pMB-MWCNTs/SPG||AOx/Hemin-MWCNTs/SPG configuration, enabling the detection of ethanol as an analytical target, both at the anodic and cathodic electrodes. Its performance was assessed by measuring polarization curves with gradually increasing ethanol concentrations ranging from 0 to 50 mM. The biosensor demonstrated a linear detection range from 0.01 to 0.3 mM, with a detection limit (LOD) of 3 ± 1 µM and a sensitivity of 64 ± 2 μW mM^−1^, with a correlation coefficient of 0.98 (RSD 8.1%, *n* = 10 electrode pairs). It exhibited robust operational stability (over 2800 s with continuous ethanol turnover) and excellent storage stability (approximately 93% of initial signal retained after 90 days). Finally, the biosensor array was integrated into a wristband and successfully evaluated for continuous alcohol abuse monitoring. This proposed system displays promising attributes for use as a flexible and wearable biosensor employing biocompatible water-based inks, offering potential applications in forensic contexts.

**Graphical Abstract:**

A novel water-based graphite ink modified with multiwalled carbon nanotubes designed for the development of a wearable self-powered biosensor enabling alcohol abuse detection through sweat analysis.

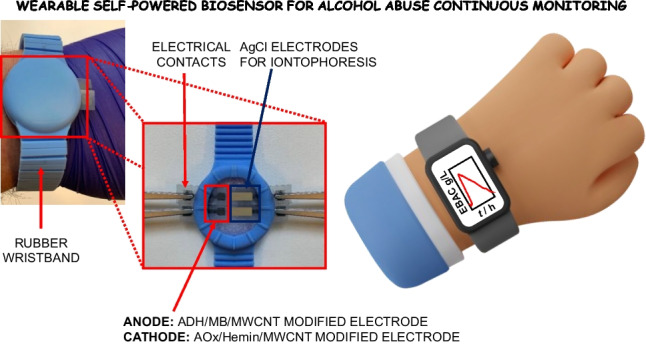

## Introduction

In recent decades, there has been a growing interest in the development of affordable wearable electrochemical biosensors for remote sensing [1–5]. This has prompted researchers to explore new technological and research solutions aimed at reducing manufacturing costs and improving the reliability, reproducibility, and stability of these biosensing platforms [6–8].

Recently, wearable self-powered biosensors enabled the development of wearable analytical sensing devices without an external electronic power source or an onboard battery, utilizing a biorecognition component to transmit sensing information [9, 10]. The ability to self-generate power is particularly crucial in situations where replacing or recharging a battery is either infeasible or impractical. While the majority of wearable self-powered biosensors primarily depend on enzymatic reactions to generate the necessary energy for their operation, there are also innovative approaches that combine multiple signal transduction techniques to both supply power and produce a detection signal simultaneously [11, 12]. Other researchers are focused on the development of more efficient energy harvesting techniques with higher power output and the creation of additional wearable self-powered biosensor devices equipped with integrated low-power wireless electronics [13, 14]. Most of enzyme-based self-powered biosensors primarily are based on a two-electrode configuration (anode and cathode), generating significant open circuit potential, substantial short circuit currents, and maximum power densities through advancements in catalysts, materials, interfaces, and cell designs [15–18].

Besides the technological advancement related to wearable self-powered devices, biosensor manufacturing constantly requires innovative materials and electrode preparation techniques to address these challenges [19–21]. While conductive inks were initially introduced several decades ago for repairing electrical circuits, they remain expensive and involve specific curing procedures that require significant time and high temperatures. Additionally, these inks are typically dispersed in organic solvents, leading to lower conductivities and potential harm to biological recognition elements such as redox enzymes, antibodies, and DNA [22–24]. The recent development of water-based lab-made inks has opened up the possibility of constructing biosensor architectures with electrochemical performance comparable to solid electrodes mentioned in existing literature [25–29].

To create reliable sensor devices, the ink mixtures must have a uniform composition with conductive properties and a moderate drying time. Rapid drying can result in surface cracking, posing challenges for electrode manufacturing, while slow drying hinders scalability and the ability to shape or size electrodes [21, 30, 31].

To ensure high reproducibility and robustness, wearable enzyme-based biosensors need to be tested under real operating conditions, taking into account factors such as the blood/tissue or peripheral bodily fluid ratio, which can be influenced by various factors like hormonal dysfunctions, sweating rate, and age [32–34]. The immobilization of bioreceptors significantly affects both reproducibility and robustness of enzyme-based amperometric biosensors [35].

Most ethanol biosensors are developed considering alcohol oxidase and nicotinamide adenine dinucleotide (NAD)–dependent alcohol dehydrogenase [36, 37]. Alcohol oxidase (AOx; alcohol:O_2_ oxidoreductase, EC 1.1.3.13) is a multi-unit enzyme composed of eight identical sub-units arranged in a quasi-cubic configuration, each containing a firmly bound cofactor, flavin adenine dinucleotide (FAD) molecule [38, 39]. Methylotrophic yeasts (such as *Hansenula*, *Pichia*, *Candida*) produce AOx in subcellular microbodies called peroxisomes while growing on methanol. AOx serves as the initial enzyme in the methanol oxidation pathway of methylotrophic yeasts. While its primary physiological function is the oxidation of methanol, it also has the capability to oxidize other short-chain alcohols like ethanol, propanol, and butanol [40]. For ethanol biosensors relying on ADH, the enzyme catalyzes the oxidation of ethanol to acetaldehyde in the presence of NAD^+^, leading to the reduction of NAD^+^ to NADH. The amperometric detection method monitors the release of electrons and protons during the oxidation of NADH. While ADH offers the advantage of being more stable and selective towards ethanol without dependence on oxygen, it faces the drawback of instability and the challenge of relying on the continuous recovery of the coenzyme NAD^+^ in the assay [41].

In addition to enzymatic detection, various analytical techniques have been suggested for identifying alcohol abuse, including chemiluminescence, high-performance liquid chromatography, and magnetic resonance spectroscopy [42]. Nonetheless, these approaches typically have limitations, such as being time-consuming, costly, and necessitating specialized laboratory equipment and trained staff. Self-powered enzyme-based biosensors offer a promising alternative, especially for creating wearable biosensors for continuous monitoring of metabolites and enabling remote healthcare [43, 44].

**Fig. 1:**
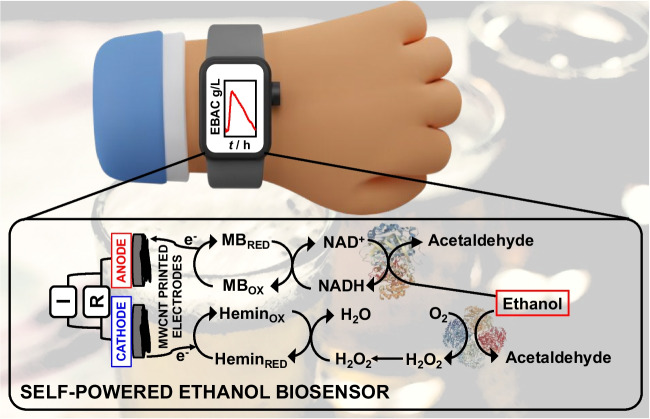
Schematic representation of self-powered wearable biosensor based on stencil-printed carbon nanotube electrodes for ethanol detection in sweat

This work reports on the formulation of a novel water-based graphite ink modified with multiwalled carbon nanotubes to fabricate the first wearable self-powered biosensor for the detection of alcohol abuse in sweat with a detection limit (LOD) of 3 ± 1 µM, a sensitivity of 64 ± 2 μW mM^−1^, and excellent storage stability (approximately 93% of initial signal retained after 90 days). The electrodes were printed onto a flexible support and modified by electrodepositing polymethylene blue (pMB) at the anode as catalyst for NADH oxidation, and hemin at the cathode as a catalyst for H_2_O_2_ reduction, as displayed in Fig. 1. Notably, alcohol dehydrogenase was further casted onto the anodic electrode, while alcohol oxidase was deposited onto the cathodic one, thus using only the analytical target to trigger the self-powered analytical detection (Fig. 1). After preliminary characterization performed in buffer and artificial sweat, the proposed array was integrated into a wrist band to continuously monitor sweat alcohol levels during daily activities with the results showing promise for future applications in remote forensic medicine. Since there is a 1:1 correspondence between blood alcohol content and sweat alcohol content, it was possible to detect alcohol abuse by triggering skin perspiration using iontophoresis [45].

## Materials and methods

### Chemicals and reagents

4-(2-Hydroxyethyl)piperazine-1-ethanesulfonic acid (HEPES), acetic acid (CH_3_COOH), d-glucose, l-lactic acid, potassium chloride (KCl), hydrochloric acid (HCl), sodium hydroxide (NaOH), uric acid, ascorbic acid, pyruvate, d-galactose, d-fructose, dopamine hydrochloride, isopropyl alcohol (IPA), graphite powder (< 20 μm, synthetic), chitosan medium molecular weight, glycerol (ACS grade ≥ 99.5%), multiwalled carbon nanotubes (MWCNTs), oxidized nicotinamide adenine dinucleotide (NAD^+^), reduced nicotinamide adenine dinucleotide (NADH), bovine serum albumin (BSA), iron(III) chloride (FeCl_3_), polyvinyl alcohol (PVA), pilocarpine nitrate, agarose, alcohol dehydrogenase (ADH) from *Saccharomyces cerevisiae*, and alcohol oxidase (AOx) from *Pichia pastoris* were purchased from Merck Millipore (formerly Sigma-Aldrich).

ADH and AOx were dissolved in 10 mM HEPES buffer pH 8.2 and 10 mM HEPES buffer at pH 7.4, respectively, with enzymatic activity of 300 U/mL for both enzymes.

All solutions were prepared using Milli-Q water (18.2 MΩ cm, Millipore, Bedford, MA, USA).

### Water-based conductive ink formulation and SPG electrode preparation

The water-based ink was developed using graphite, chitosan, and glycerol as the conductive material, binder, and stabilizer, respectively. A 2.5% w/v chitosan solution was prepared by dissolving chitosan in 1 M acetic acid, which was then stirred overnight at room temperature. The chitosan solution was subsequently diluted to 1% w/v with distilled water, resulting in a final acetic acid concentration of 0.4 M. The conductive ink was prepared by mixing 5.4 g of graphite powder with 9 mL of the previously prepared chitosan solution and 300 µL of glycerol, as previously optimized [22].

Next, the stencil-printed graphite (SPG) electrodes were fabricated using polyethylene terephthalate (PET) sheets that were cleaned three times with IPA and distilled water, then sanded with fine emery paper (1500 grit) to enhance ink adhesion. A stencil was created on a Smart Vinyl adhesive sheet using a Cricut Explore® 3 equipped with Design Space Software v.7.3.95. After applying the stencil to the PET sheet, 500 µL of the conductive ink was placed onto the PET sheet and spread evenly with a scraper. The prepared electrode was allowed to dry at room temperature for 10 min and then cured in an oven at 100 °C for 1 min. Subsequently, the stencil was removed, and the connecting track between the working electrode and the pad was insulated with nail polish [21].

### Anode electrode preparation

To prepare ADH/pMB-MWCNTs/SPG, 3 μL of a MWCNTs EtOH:H_2_O suspension (70:30, 10 mg mL^−1^), previously ultrasonicated for 1 week, was drop-cast onto the electrode and allowed to dry at room temperature. Afterwards, the so prepared MWCNTs/SPG were further modified by electrodeposition of pMB sweeping the potential between − 0.4 and + 1.2 V vs. Ag/AgCl in 0.25 mM MB solution (0.02 M borate buffer pH 9.14, supporting electrolyte 0.1 M KNO_3_, sweep rate 50 mV s^−1^, from 5 to 35 cycles) [17]. Alternatively, the electrodeposition of pMB was performed by using a potentiostatic pulsed method, adopting the following parameters: + 1.2 V oxidation potential and 1 s oxidation pulse time, − 0.4 V reduction potential, 1 s reduction pulse time, and 0.2 s as interval time, ranging the total number of cycles from 50 to 350 cycles. The electrodeposition was performed in 0.25 mM MB solution (0.02 M borate buffer pH 9.14, supporting electrolyte 0.1 M KNO_3_). Afterwards, the pMB-MWCNTs/SPG electrodes were modified with 5 µL of ADH. Finally, 5 µL of a BSA solution (10 mg mL^−1^) was deposited onto the electrode to prevent the electrode biofouling and interferences. The electrodes were further conditioned overnight in 10 mM HEPES buffer pH 8.2 at + 4 °C.

### Cathode electrode preparation

To prepare AOx/Hemin-MWCNTs/SPG, 3 μL of a MWCNTs EtOH:H_2_O suspension (70:30, 10 mg mL^−1^), previously ultrasonicated for 1 week, was drop-cast onto the electrode and allowed to dry at room temperature [46]. Afterwards, the so prepared MWCNTs/SPG were further modified by drop-casting 5 µL of Hemin solution (1 mg mL^−1^ in DMF) and allowed to dry at room temperature [47, 48]. Afterwards, the Hemin-MWCNTs/SPG electrodes were modified with 5 µL of AOx. Finally, 5 µL of a BSA solution (10 mg mL^−1^) was deposited onto the electrode to prevent the electrode biofouling and interferences. The electrodes were further conditioned overnight in 10 mM HEPES buffer pH 7.4 at + 4 °C.

### Iontophoretic electrode preparation and protocol

The iontophoretic electrodes were obtained by stencil-printing LOCTITE® ECI 1010 E&C silver ink with a geometric area of 2 cm^2^ and square shape measuring 20 × 10 mm and cured according to manufacturing instructions [49]. Both electrodes were chlorinized by using 0.1 M FeCl_3_ solution for 30 s. After cutting, the anode electrode was covered with an anodic iontophoretic hydrogel containing 4% agarose gel, 0.1% oxidized nicotinamide adenine dinucleotide (NAD^+^), and 2% pilocarpine, while the cathode electrode was situated 0.2 cm away from the anode and coated with a cathodic hydrogel comprising 5% PVA and 1% potassium nitrate. The iontophoresis protocol was optimized to generate sweat volume and ensure skin response to iontophoretic current. Sweat generation was achieved by delivering pilocarpine across the skin using an optimized electrical current density of approximately 0.4 mA cm^−2^ flowing from the anodic to the cathodic compartment for 10 min.

### Equipment and measurements

Cyclic voltammetry (CV), chronoamperometry, open circuit potential (OCP), and linear sweep voltammetry (LSV, polarization curves) experiments were conducted using a PalmSens4 electrochemical workstation equipped with PSTrace 5.6v software. All potentials were referenced to a BASi Ag|AgCl|KCl electrode (all potential values are reported in the paper considering this reference) and a platinum wire was used as the reference and counter electrode, respectively. Stencil-printed graphite (SPG) electrodes with a geometric area of 9 mm^2^ and square shape measuring 3 × 3 mm were employed as the working electrodes. Polarization curves were recorded using linear sweep voltammetry (LSV) in potentiostatic mode at a scan rate of 1 mV s^−1^, ranging from the open circuit voltage (OCV) to 0 V. The bioanode, ADH/pMB-MWCNTs/SPG, was employed as the working electrode, while AOx/Hemin-MWCNTs/SPG served as a combined reference and counter electrode. The polarization curves were recorded in 10 mM HEPES pH 7 with different ethanol concentrations (10, 50, 100, 150, 250, 300, 500, 1000, 2500, 5000, 25,000, and 50,000 µM). The temperature-controlled experiments were conducted using a cryostatic bath (LAUDA RM6, Delran, NJ, USA) with a precision of ± 0.01 °C.

Morphological characterization was carried out using a field emission scanning electron microscope (FE-SEM), model Σigma Zeiss (Jena, Germany). The images were captured using the in-lens detector, with a 5-kV acceleration voltage, 4-mm working distance, and 30-µm aperture, in top-view mode without any additional sample treatment.

### Volunteer for experiments in sweat

Three male and three female volunteers, aged 30 and seemingly in good health, participated in all measurements and procedures described herein, posing minimal health risks. Written informed consent was obtained from the volunteers. Personal data handling adhered to the regulations outlined in the GDPR law 675/1996, in line with Directive 95/46/EC, aimed at safeguarding personal integrity during data processing. The research project and data collection present no risk of harm, and all information collected is publicly available.

## Results and discussion

### Electrochemical and SEM characterization of pMB modified MWCNTs/SPG electrodes

**Fig. 2:**
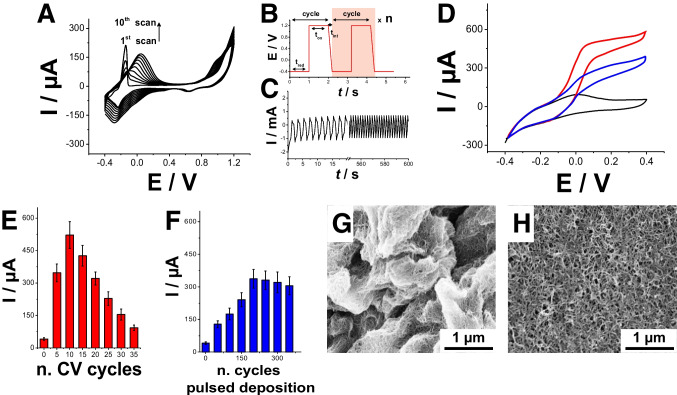
**A** CVs for pMB electropolymerization performed onto MWCNTs/SPG sweeping the potential between − 0.4 and + 1.2 V vs. Ag/AgCl in 0.25 mM MB solution (0.02 M borate buffer pH 9.14, supporting electrolyte 0.1 M KNO_3_, scan rate 50 mV s^−1^—10 cycles). **B** Scheme of the potentiostatic pulsed electrodeposition protocol. **C** Potentiostatic pulsed electropolymerization performed onto MWCNTs/SPG in 0.25 mM MB solution (0.02 M borate buffer pH 9.14, supporting electrolyte 0.1 M KNO_3_). **D** CVs for pMB-MWCNTs/SPG electrode in non-turnover (10 mM HEPES buffer pH = 7.2 + 100 mM KCl, black curve) and turnover conditions (addition of 1 mM NADH, red curve, electropolymerized by CV—blue curve, electropolymerized by potentiostatic pulsed method), scan rate 5 mV s^−1^. **E** Bar diagram for pMB electropolymerization by cyclic voltammetry performed onto MWCNTs/SPG sweeping the potential between − 0.4 and + 1.2 V vs. Ag/AgCl in 0.25 mM MB solution (0.02 M borate buffer pH 9.14, supporting electrolyte 0.1 M KNO_3_, scan rate 50 mV s^−^.^1^) from 0 to 35 cycles. **F** Bar diagram for pMB electropolymerization by potentiostatic pulsed method onto MWCNTs in 0.25 mM MB solution (0.02 M borate buffer pH 9.14, supporting electrolyte 0.1 M KNO_3_) from 0 to 350 cycles. **G** Scanning electron microscopy (SEM) image of pMB-MWCNTs/SPG obtained by CV (10 cycles). **H** Scanning electron microscopy (SEM) image of pMB-MWCNTs/SPG obtained by potentiostatic pulsed method (200 cycles)

The influence of the redox mediator on the analytical performance of the anodic electrode was investigated by electrodepositing pMB by cyclic voltammetry and potentiostatic pulse method. Notably, Fig. 2A reports CVs for pMB electrodeposition performed by scanning SPG electrodes between − 0.4 and + 1.2 V at 50 mV s^−1^ as scan rate (10 cycles). Two redox peaks are observed: a cathodic peak at − 0.22 V and an anodic peak at − 0.20 V in curve 1. These redox peaks are attributed to the reduction and oxidation of methylene blue. The current of the anodic peak at − 0.20 V decreases rapidly, and a new anodic peak emerges at approximately − 0.06 V in the second cycle, with its current increasing gradually over subsequent potential cycles. The potential of this latter peak shifts to 0.10 V by the seventh cycle. Following electrolysis, a thin film with a greenish hue is observed on the working SPG electrode [50–52]. Alternatively, the potentiostatic pulse method was adopted as described in Fig. 2B. The electrodeposition of pMB was conducted using the following parameters: an oxidation potential of + 1.2 V and an oxidation pulse duration of 1 s, a reduction potential of − 0.4 V, a reduction pulse duration of 1 s, and an interval time of 0.2 s in both steps. Figure 2C reports the potentiostatic pulsed electrodeposition for 250 cycles, where the pulses achieved steady-state current values after 8 cycles probably due pMB homogeneous surface coverage (additional cycles lead to multilayer deposition and increased nanostructuration). Both types of anodic electrodes, namely pMB-MWCNTs/SPG obtained with cyclic voltammetry and potentiostatic pulsed method, were investigated in 10 mM HEPES buffer pH 7.2 (containing 100 mM KCl as supporting electrolyte) as non-turnover experiment, and in the presence of 1 mM NADH to evaluate their catalytic efficiency (Fig. 2D). Notably, pM-MWCNTs/SPG electrodes obtained by cyclic voltammetry and potentiostatic pulsed method reported a similar CV in 10 mM HEPES buffer pH 7.2 (containing 0.1 M KCl), highlighting the presence of a couple of redox peaks with a formal potential (E^0^′) of − 0.06 V (black curve). After adding 1 mM NADH, pM-MWCNTs/SPG obtained by cyclic voltammetry (red curve, modified with 10 electrodeposition cycles) and potentiostatic pulsed method (blue curve, 200 potentiostatic pulsed cycles) reported a catalytic current (I_cat_) of 524 ± 23 µA and 342 ± 18 µA at + 0.2 V, respectively, probably due to higher polymer surface coverage.

Furthermore, both electrodeposition methods were optimized keeping constant oxidation/reduction potentials and times, as reported in the literature, but varying the number of CVs (Fig. 2E) and potentiostatic pulsed method (Fig. 2F) [53]. Notably, pMB-MWCNTs/SPG electrodes obtained by cyclic voltammetry showed the highest catalytic current for NADH oxidation after 10 cycles (524 ± 23 µA). Notably, the catalytic current was decreasing with the number of electrodeposition cycles (112 ± 9 µA with 35 cycles). This can be ascribed to pMB multilayer formation that might not be electrically connected with the electrode surface. pMB-MWCNTs/SPG electrodes obtained by the potentiostatic pulsed method showed a similar trend with the highest catalytic current for NADH oxidation at 200 cycles (342 ± 18 µA). The optimized pMB-MWCNTs/SPG electrodes obtained by cyclic voltammetry (10 cycles) were further characterized by scanning electron microscopy (SEM) observing a bundle-like structure covered by pMB layers (Fig. 2G). Moreover, the optimized pMB-MWCNTs/SPG electrodes obtained by potentiostatic pulsed method (200 cycles) showed a homogeneously dispersed layer of MWCNTs covered by pMB layers. The different morphology can be ascribed to the different electrodeposition methods that led to MWCNTs reorganization onto the electrode surface and different pMB layers growth process. Hence, the optimized pMB-MWCNTs/SPG electrodes obtained by cyclic voltammetry (10 cycles) were selected as potential bioanode electrodes for the development of ethanol self-powered biosensor.

### Electrochemical characterization of bioanode ADH/pMB-MWCNTs/SPG

**Fig. 3:**
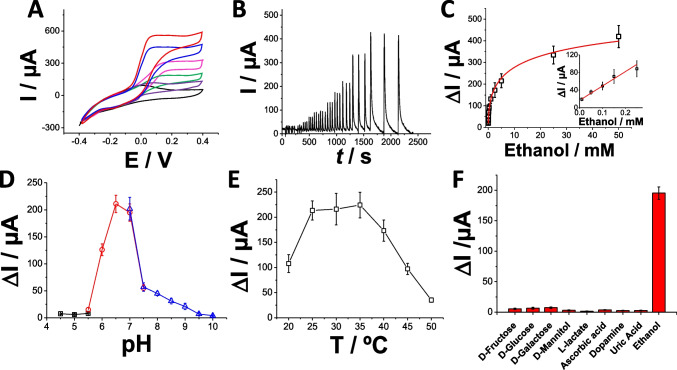
**A** CVs for ADH/pMB-MWCNTs/SPG electrode in non-turnover (10 mM HEPES buffer pH = 7.2 + 100 mM KCl + 1 mM NAD^+^, black curve) and turnover conditions (addition of 5, 10, 15, 20, and 50 mM ethanol, represented by purple, green, magenta, blue, and red curves, respectively), scan rate 5 mV s^−1^. **B** Chronoamperometry at E_appl_: + 0.2 V and 0.5 mL min^−1^ as flow rate by increasing substrate concentration in the range 0–50 mM for ethanol (10 mM HEPES buffer pH = 7.2 + 100 mM KCl + 1 mM NAD^+^). **C** Calibration curve for ADH/pMB-MWCNTs/SPG electrode in 10 mM HEPES buffer pH = 7.2 + 100 mM KCl + 1 mM NAD^+^ based on the chronoamperometric measurements performed at E_appl_: + 0.2 V by increasing substrate concentration in the range 0–50 mM for ethanol—inset: linear range of calibration curve for ADH/pMB-MWCNTs/SPG electrode. Effect of different pHs (**D**) and temperature (**E**) on ADH/pMB-MWCNTs/SPG electrode: 10 mM acetate buffer (black), 10 mM MOPS buffer (red), 10 mM HEPES buffer (blue). Experimental conditions: 5 mM ethanol, applied potential + 0.2 V, and flow rate 0.5 mL min^−1^. **F** Influence of interfering compounds on ethanol response: 200 µM ascorbic acid, 200 µM uric acid, 200 µM d-glucose, 200 µM pyruvate, 200 µM d-fructose, 200 µM dopamine, 200 µM uric acid, and 5 mM ethanol measured with chronoamperometry at E_appl_: + 0.2 V and flow rate 0.5 mL min.^−1^

The pMB-MWCNTs/SPG electrodes, prepared as described, were further modified with alcohol dehydrogenase (ADH) from *Saccharomyces cerevisiae* to create second-generation ethanol biosensors. In Fig. 3A, cyclic voltammograms (CVs) are depicted under both non-turnover (black curve) and turnover conditions (with the addition of 5, 10, 15, 20, and 50 mM ethanol, represented by purple, green, magenta, blue, and red curves, respectively). Under non-turnover conditions, the ADH/pMB-MWCNTs/SPG electrodes exhibited a pair of redox peaks with a formal potential E^0^′ =  − 0.067 V attributed to the pMB layer deposited onto MWCNTs/SPG electrodes (Fig. 3A, black curve). Upon substrate addition, a significant, mass-transfer-limited electrocatalytic response commencing at E_ONSET_ =  − 0.08 V with a maximum current of 112 µA (5 mM ethanol, purple curve), 174 µA (10 mM ethanol, purple curve), 307 µA (15 mM ethanol, purple curve), 434 µA (20 mM ethanol, purple curve), and 518 µA (50 mM ethanol, purple curve) at E =  + 0.2 V was observed (Fig. 3A, purple, green, magenta, blue, and red curves, respectively). The limitation in mass transfer is associated with the formation of bundles consisting of pMB-MWCNTs, which improves the catalytic efficiency for NADH oxidation. Meanwhile, the roughness and porosity of SPG electrodes regulate the diffusion occurring at the electrode surface.

The modified electrodes underwent chronoamperometry testing by incrementally increasing substrate concentration within the range of 0–50 mM for ethanol (Fig. 3B).

The calibration curve derived from the chronoamperometric response of the ADH/pMB-MWCNTs/SPG electrodes (spanning overall the 1 × 10^−5^–5 × 10^−2^ M), as depicted in Fig. 3C, revealed a linear range from 0.01 to 0.3 mM (Fig. 3C, inset), with a detection limit (LOD) of 3 ± 1 µM (calculated using LOD = 3.3 × (σ/S) where σ represents the absolute standard deviation of the intercept, and S denotes the slope of the calibration curve), and a sensitivity of 326 ± 28 μA mM^−1^, accompanied by a correlation coefficient of 0.99 (RSD 7.2%, *n* = 10). Furthermore, the calibration curve was fitted to ascertain the classical Michaelis–Menten kinetic parameters, resulting in an I_max_ of 653 ± 24 µA and an apparent Michaelis–Menten constant (K_M_^app^) of 19 ± 4 mM (approximately 200 times higher than K_M_ measured in solution) [54]. This discrepancy could be attributed to the controlled diffusion of enzymatic product (i.e., NADH) through the rough electrode surface, typically leading to an extended linear range. However, the presence of pMB-MWCNTs bundles may have impeded their availability for surface catalytic reactions, where the enzyme is adsorbed. The analytical performance of the proposed electrode platform could be attributed to the low quantity of enzyme effectively immobilized onto the electrode surface and the limited accessibility of the pMB layer for catalytic NADH oxidation. The influence of pH and temperature on the performance of ADH/pMB-MWCNTs/SPG electrodes was investigated. To cover a broad pH spectrum from 4.5 to 10, three different buffers, namely acetate, MOPS, and HEPES, were utilized. The current signal exhibited an upward trend with increasing pH, peaking at pH 6.5 (218 ± 11 µA), and subsequently declining as the pH surpassed 6.5 (Fig. 3D). Similarly, the impact of temperature was evaluated in 10 mM HEPES buffer at pH 6.5 (containing 100 mM KCl). The findings revealed an optimal temperature range of 25–35 °C for ADH/pMB-MWCNTs/SPG electrodes (Fig. 3E), consistent with previously reported literature. The selectivity of the ADH/pMB-MWCNTs/SPG electrodes was assessed to gauge the effect of potential interfering compounds on their response (Fig. 3F). The signal obtained at a fixed ethanol concentration was compared with signals obtained with equivalent quantities of various potential interfering compounds, including d-fructose, d-galactose, d-mannitol, d-glucose, l-lactate, ascorbic acid, uric acid, and dopamine. The outcomes indicated that there was no noteworthy current response for the potential interfering compounds due to the presence of a BSA protecting layer mainly acting as diffusional barrier and its isoelectric point.

### Electrochemical characterization of biocathode AOx/Hemin-MWCNTs/SPG

**Fig. 4:**
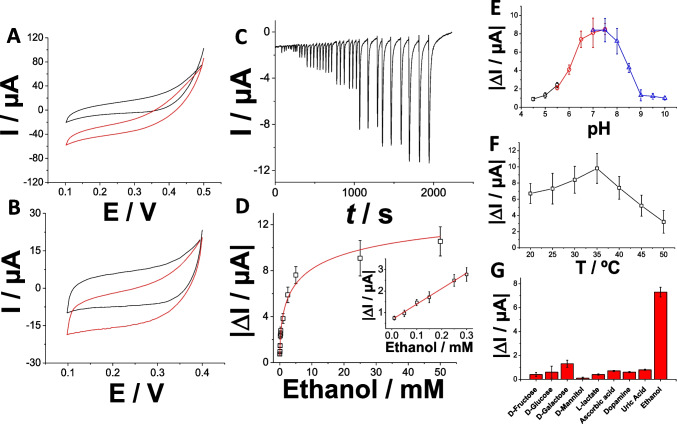
**A** CVs for Hemin-MWCNTs/SPG electrode in non-turnover (10 mM HEPES buffer pH = 7.2 + 100 mM KCl, black curve) and turnover conditions (addition of 1 mM H_2_O_2_), scan rate 5 mV s^−1^. **B** CVs for AOx/Hemin-MWCNTs/SPG electrode in non-turnover (10 mM HEPES buffer pH = 7.2 + 100 mM KCl, black curve) and turnover conditions (addition of 10 mM ethanol), scan rate 5 mV s^−1^. **C** Chronoamperometry at E_appl_: + 0.2 V and 0.5 mL min^−1^ as flow rate by increasing substrate concentration in the range 0–50 mM for ethanol (10 mM HEPES buffer pH = 7.2 + 100 mM KCl). **D** Calibration curve for AOx/Hemin-MWCNTs/SPG electrode in 10 mM HEPES buffer pH = 7.2 + 100 mM KCl based on the chronoamperometric measurements performed at E_appl_: + 0.2 V by increasing substrate concentration in the range 0–50 mM for ethanol—inset: linear range of calibration curve for AOx/Hemin-MWCNTs/SPG electrode. Effect of different pHs (**E**) and temperature (**F**) on AOx/Hemin-MWCNTs/SPG electrode: 10 mM acetate buffer (black), 10 mM MOPS buffer (red), 10 mM HEPES buffer (blue). Experimental conditions: 5 mM ethanol, applied potential + 0.2 V, and flow rate 0.5 mL min^−1^. **G** Influence of interfering compounds on ethanol response: 200 µM ascorbic acid, 200 µM uric acid, 200 µM d-glucose, 200 µM pyruvate, 200 µM d-fructose, 200 µM dopamine, 200 µM uric acid, and 5 mM ethanol measured with chronoamperometry at E_appl_: + 0.2 V and flow rate 0.5 mL min.^−1^

Moreover, Hemin-MWCNTs/SPG electrodes were investigated in 10 mM HEPES buffer pH 7.2 (containing 100 mM KCl as supporting electrolyte) as non-turnover condition, and in the presence of 1 mM H_2_O_2_ to evaluate their catalytic efficiency towards H_2_O_2_ reduction to H_2_O (Fig. 4A). Particularly, Hemin-MWCNTs/SPG electrodes showed a CV in 10 mM HEPES buffer pH 7.2 (containing 0.1 M KCl), without reporting any redox wave (black curve). After adding 1 mM H_2_O_2_, Hemin-MWCNTs/SPG electrodes reported a catalytic current (I_cat_) of − 58 ± 6 µA at E =  + 0.1 V. The Hemin-MWCNTs/SPG electrodes, prepared as described, were further modified with alcohol oxidase (AOx) from *Pichia pastoris* to create first-generation ethanol biosensors able to work as biocathode in an enzymatic fuel cell due to the high operational voltage of Hemin as catalyst for H_2_O_2_ reduction [47, 48]. In Fig. 4B, cyclic voltammograms (CVs) are depicted under both non-turnover (black curve) and turnover conditions (with the addition of 10 mM ethanol, red curve). Under non-turnover conditions, the AOx/Hemin-MWCNTs/SPG electrodes did not exhibit any redox wave (Fig. 4B, black curve). Upon addition of 10 mM ethanol, a catalytic response commencing at E_ONSET_ =  + 0.36 V with a maximum current of − 19 ± 2 µA at E =  + 0.1 V was observed (Fig. 4B, red curve).

The modified electrodes were subjected to chronoamperometry testing by gradually increasing substrate concentration within the range of 0–50 mM for ethanol (Fig. 4C). The calibration curve derived from the chronoamperometric response of the AOx/Hemin-MWCNTs/SPG electrodes (spanning overall the 1 × 10^−5^–5 × 10^−2^ M), as illustrated in Fig. 4D, showed a linear range from 0.01 to 0.3 mM (Fig. 4D, inset), with a detection limit (LOD) of 3 ± 1 µM, and a sensitivity of 7.2 ± 0.3 μA mM^−1^, accompanied by a correlation coefficient of 0.99 (RSD 5.8%, *n* = 10). Additionally, the calibration curve was fitted to determine the classical Michaelis–Menten kinetic parameters, resulting in an I_max_ of 16 ± 2 µA and an apparent Michaelis–Menten constant (K_M_^app^) of 8.2 ± 0.9 mM, similar to other K_M_^app^ data reported in the literature. The analytical performance of the proposed electrode platform could be attributed to the intimate contact between the enzyme and the catalyst specifically acting towards H_2_O_2_ reduction. The impact of pH and temperature on the performance of AOx/Hemin-MWCNTs/SPG electrodes was examined. To cover a wide pH range from 4.5 to 10, three different buffers, namely acetate, MOPS, and HEPES, were employed. The current signal exhibited an upward trend with increasing pH, peaking at pH 7.5 (8.3 ± 0.7 µA), and subsequently declining as the pH surpassed 7.5 (Fig. 4E). Similarly, the effect of temperature was assessed in 10 mM HEPES buffer at pH 7.5 (containing 100 mM KCl). The findings indicated an optimal temperature of 35 °C for AOx/Hemin-MWCNTs/SPG electrodes (Fig. 4F), consistent with previously reported literature [55]. The selectivity of the AOx/Hemin-MWCNTs/SPG electrodes was evaluated to assess the influence of potential interfering compounds on their response (Fig. 4G). The signal obtained at a fixed ethanol concentration was compared with signals obtained with equivalent amounts of various potential interfering compounds, including d-fructose, d-galactose, d-mannitol, d-glucose, l-lactate, ascorbic acid, uric acid, and dopamine. The results indicated no significant current response for the potential interfering compounds due to the presence of a BSA protecting layer mainly acting as diffusional barrier and its isoelectric point [56].

### Characterization of enzymatic fuel cell as ethanol self-powered biosensor

**Fig. 5:**
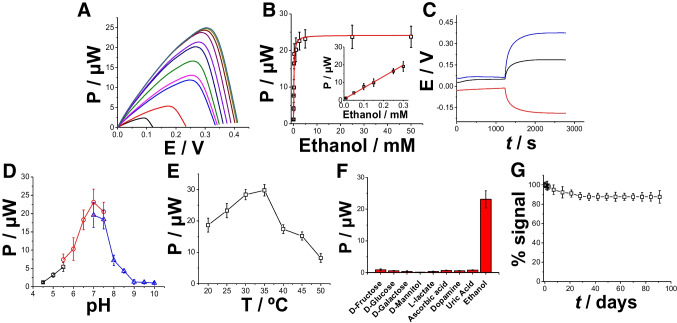
**A** Polarization curves for ADH/pMB-MWCNTs/SPG||AOx/Hemin-MWCNTs/SPG EFC by increasing substrate concentration in the range 0–50 mM for ethanol (10 mM HEPES buffer pH = 7 + 100 mM KCl + 1 mM NAD^+^), LSV at scan rate 1 mV s^−1^. **B** Calibration curve for ADH/pMB-MWCNTs/SPG||AOx/Hemin-MWCNTs/SPG EFC in 10 mM HEPES buffer pH = 7 + 100 mM KCl + 1 mM NAD^+^ based on the polarization curves recorded by increasing substrate concentration in the range 0–50 mM for ethanol—inset: linear range of calibration curve for ADH/pMB-MWCNTs/SPG||AOx/Hemin-MWCNTs/SPG EFC. **C** ADH/pMB-MWCNTs/SPG bioanode OCP (red line), AOx/Hemin-MWCNTs/SPG biocathode (black line), and OCV measurements for a single EFC (blue line) operating continuously over 2800 in non-turnover (10 mM HEPES buffer pH = 7 + 100 mM KCl + 1 mM NAD^+^, black curve) and turnover conditions (addition of 5 mM ethanol). Effect of different pHs (**D**) and temperature (**E**) on ADH/pMB-MWCNTs/SPG||AOx/Hemin-MWCNTs/SPG EFC: 10 mM acetate buffer (black), 10 mM MOPS buffer (red), 10 mM HEPES buffer (blue). Experimental conditions: 5 mM ethanol, applied potential + 0.2 V, and flow rate 0.5 mL min^−1^. **F** Influence of interfering compounds on ethanol response: 200 µM ascorbic acid, 200 µM uric acid, 200 µM d-glucose, 200 µM pyruvate, 200 µM d-fructose, 200 µM dopamine, 200 µM uric acid, and 5 mM ethanol measured with polarization curves. **G** Storage stability of ADH/pMB-MWCNTs/SPG||AOx/Hemin-MWCNTs/SPG EFC

Finally, the selected bioelectrodes, ADH/pMB-MWCNTs/SPG and AOx/Hemin-MWCNTs/SPG, were assembled as bioanode (connected as the working electrode) and biocathode (connected as the reference-counter electrode) to obtain an enzymatic fuel cell (EFC) working as an ethanol self-powered biosensor [57]. The EFC was characterized by running polarization curves gradually increasing substrate concentration within the range of 0–50 mM for ethanol (Fig. 5A). ADH/pMB-MWCNTs/SPG||AOx/Hemin-MWCNTs/SPG EFC reported an OCV of 0.052 ± 0.007 V with a power output of 0.3 ± 0.1 µW at 0.044 V in non-turnover conditions (not shown) and 0.42 ± 0.004 V with a power output of 25.4 ± 1.1 µW at 0.33 V (green curve, 50 mM ethanol). The calibration curve derived from the LSV curves of the AOx/Hemin-ADH/pMB-MWCNTs/SPG||AOx/Hemin-MWCNTs/SPG EFC (spanning overall the 1 × 10^−5^–5 × 10^−2^ M), as illustrated in Fig. 5B, showed a linear range from 0.01 to 0.3 mM (Fig. 5B, inset), with a detection limit (LOD) of 3 ± 1 µM, and a sensitivity of 64 ± 2 μW mM^−1^, accompanied by a correlation coefficient of 0.98 (RSD 8.1%, *n* = 10 couple of electrodes).

Both the anode and cathode electrodes, namely ADH/pMB-MWCNTs/SPG and AOx/Hemin-MWCNTs/SPG, were characterized by open circuit potentiometry (OCP) to assess their individual operational stability over 2800 s, as shown in Fig. 5C (anode: red curve and cathode: black curve). After the addition of 5 mM ethanol as substrate, ADH/pMB-MWCNTs/SPG electrodes showed an OCP of − 0.168 ± 0.008 V and AOx/Hemin-MWCNTs/SPG electrodes showed an OCP of 0.341 ± 0.004 V, displaying an increased stability over 2800 s, probably due to the porosity of the electrodes, which affects the amount of enzyme molecules immobilized and its stability. After assembling the EFC, the OCV resulted to be 0.516 ± 0.002 V (blue curve), which is in good agreement with the OCV that can be calculated as a difference between the OCP curves measured individually, namely 0.509 V.

The impact of pH and temperature on the performance of ADH/pMB-MWCNTs/SPG||AOx/Hemin-MWCNTs/SPG EFC was examined. To cover a wide pH range from 4.5 to 10, three different buffers, namely acetate, MOPS, and HEPES, were employed. The power output signal exhibited an upward trend with increasing pH, peaking at pH 7 (24.3 ± 1.7 µW), and subsequently declining as the pH surpassed 7 (Fig. 5D). Similarly, the effect of temperature was assessed in 10 mM HEPES buffer at pH 7 (containing 100 mM KCl). The findings indicated an optimal temperature of 35 °C for ADH/pMB-MWCNTs/SPG||AOx/Hemin-MWCNTs/SPG EFC (Fig. 5E), consistent with previously reported literature [57]. The selectivity of the ADH/pMB-MWCNTs/SPG||AOx/Hemin-MWCNTs/SPG EFC was evaluated to assess the influence of potential interfering compounds on their response (Fig. 5F). The signal obtained at a fixed ethanol concentration was compared with signals obtained with equivalent amounts of various potential interfering compounds, including d-fructose, d-galactose, d-mannitol, d-glucose, l-lactate, ascorbic acid, uric acid, and dopamine. The results indicated no significant current response for the potential interfering compounds due to the presence of a BSA protecting layer mainly acting as diffusional barrier and its isoelectric point [56]. Besides the preliminary analytical features, the storage stability of the proposed platforms was tested by recording the power output response for 20 consecutive measurements every day over a period of 90 days. The stability measurements were performed by continuously supplying 5 mM ethanol (Fig. 5G) dispensed through a FIA system. In particular, ADH/pMB-MWCNTs/SPG||AOx/Hemin-MWCNTs/SPG EFC reported a response drop of its initial signal of 7% after 25 days, probably because of the enzyme intrinsic stability and the porosity/roughness of SPG electrodes, which is stabilizing the enzymatic layer. Table 1 summarizes the results obtained for other self-powered ethanol biosensors. In this context, one must also take into account the variability in composition observed in peripheral bodily fluids, especially concerning electrolytes, which could impact the consistency of sweat analyses. Other platforms reported wider linear ranges (mM concentrations) but higher LOD compared to the platform herein proposed [57–61]. The proposed self-powered biosensor was integrated within a wristband and integrated with a system to induce/control the perspiration rate and applying a Nafion membrane to control the diffusion at the electrode surface reporting a LOD of 0.1 g/L and the linear range to 0.5–14 g/L that enables the quantification at the threshold EBAC in most of the countries in the world. In this regard, the proposed biosensor showed higher LOD but wider linear range compared with other platforms previously reported probably due to the integration of a iontophoretic system to induce/control the sweating rate [62–64].

**Table 1:** Comparison with other self-powered ethanol biosensors reported in literature. *List of abbreviations*: *TEMPO* 2,2,6,6-tetramethylpiperidinyl-N-oxyl, *ADH* alcohol dehydrogenase, *AOx* alcohol oxidase, *C* carbon electrode, *G* graphite electrode, *HRP* horseradish peroxidase, *MP-8* microperoxidase-8, *MWCNTs* multiwalled carbon nanotubes, *OxDc* oxalate decarboxylase enzyme, *Pt* platinum, *pMB* polymethylene blue, *pMG* polymethylene green, *Pyr* pyrrole, *QH-ADH* pyrroloquinoline quinone–dependent alcohol dehydrogenase, *SPG* stencil-printed graphite electrode, *TB* toluidine blue

Electrode platform	Linear range, mM	LOD, µM	Ref
QH-ADH/G||AOx/MP-8/G	0.1–2	-	[58]
ADH/pMG-Pyr/MWCNTs/G||Pt/C	0.1–5	~ 30	[59]
MWCNT-COOH/pyrene-TEMPO/OxDc||Pt/C	0.1–5	~ 30	[60]
ADH/TB-MWCNTs/G||AOx/HRP-MWCNTs/G	0.1–1	~ 30	[61]
ADH/pMB-MWCNTs/SPG||AOx/Hemin-MWCNTs/SPG	0.01–0.3	3	This work

### Alcohol self-powered biosensor integrated in a wrist band

**Fig. 6:**
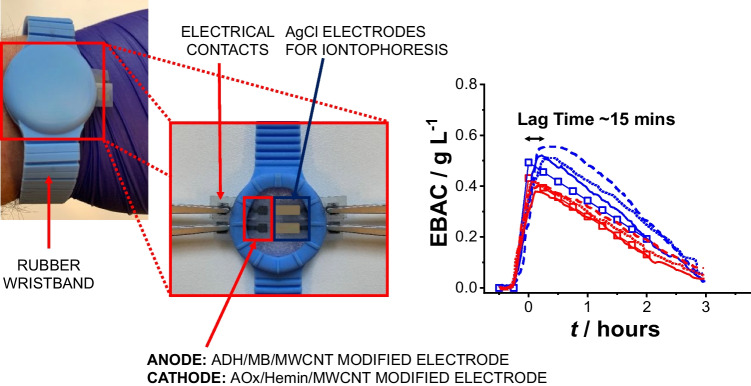
ADH/pMB-MWCNTs/SPG||AOx/Hemin-MWCNTs/SPG self-powered biosensor and iontophoretic electrodes to indue skin perspiration enclosed in the wrist band; EBAC measurements performed by using ADH/pMB-MWCNTs/SPG||AOx/Hemin-MWCNTs/SPG self-powered biosensor (blue curves for healthy male volunteers and red curves for healthy female volunteers) for the continuous monitoring of alcohol abuse in terms of power output; calculated EBAC based on Widmark formula (blue scattered curve for healthy male volunteers and red scattered curve for healthy female volunteers)

After preliminary analytical characterization of both bioanode and biocathode electrodes separately and assembled as EFC, they were integrated within a wristband to perform continuous alcohol abuse monitoring in sweat. As shown in Fig. 6, both ADH/pMB-MWCNTs/SPG and AOx/Hemin-MWCNTs/SPG electrodes were placed within the rubber wrist band together with printed silver AgCl electrodes to induce skin perspiration through iontophoretic process. The recess within the rubber wristband created an electrochemical cell with a thickness of 2 mm, which enabled sweat accumulation. The wrist band was worn by three voluntary healthy male and female patients.

Figure 6 shows the power output recording for ethanol detection (red curve) in sweat measured for three healthy male volunteers (blue curve) and three healthy female volunteers (red curve), which was correlated with estimated blood alcohol content in g L^−1^ (scatter point black lines). A method formulated by Swedish professor Erik Widmark in the 1920s can be utilized to approximate the estimated blood alcohol content (EBAC) [65]. This was calculated using Eq. 1:

1 $$\text{EBAC}=\frac{A}{{V}_{d}}-\beta T$$

where *A* represents the quantity of alcohol ingested (in g), *T* signifies the duration for which alcohol remained in the bloodstream (typically the time elapsed since consumption commenced), measured in hours, *β* denotes the elimination rate of alcohol, usually averaging about 0.15 g/L/h, and *V*_*d*_ indicates the volume of distribution (in L), typically calculated as body weight (in kg) multiplied by 0.71 L/kg for males and 0.58 L/kg for females. Considering three healthy volunteer male patients with a weight of approximately 80 kg and drinking a total of 28 g of alcohol, the EBAC profile showed a maximum of 0.50 g L^−1^ decreasing over 2 h (black scattered curve). Similarly, three healthy volunteer female patients with a weight of approximately 60 kg and drinking a total of 15 g of alcohol, the EBAC profile showed a maximum of 0.43 g L^−1^ decreasing over 2 h (black scattered curve). A lag time of 15 min was observed in sweat EBAC output recording probably due to the activation of iontophoretic process and metabolites transport within the peripheral blood microcirculation system [45].

## Conclusions

In this study the first example of self-powered ethanol biosensor based on water-based graphite inks modified with MWCNTs and using ethanol as input for both EFC compartments (bioanode and biocathode) was reported. The self-powered biosensor was assembled using ADH/pMB-MWCNTs/SPG||AOx/Hemin-MWCNTs/SPG. The self-powered biosensor was characterized by running polarization curves gradually increasing substrate concentration within the range of 0–50 mM for ethanol, reporting a linear range from 0.01 to 0.3 mM, with a detection limit (LOD) of 3 ± 1 µM, and a sensitivity of 64 ± 2 μW mM^−1^, accompanied by a correlation coefficient of 0.98 (RSD 8.1%, *n* = 10 couple of electrodes). The biodevice showed a good operational stability (over 2800 s with continuous ethanol turnover) and an excellent storage stability (~ 93% of initial signal retained after 90 days). Finally, the proposed array was integrated in a wristband and successfully tested for the continuous monitoring of alcohol abuse.

The suggested system exhibits encouraging characteristics for utilization as a versatile and wearable biosensor using biocompatible water-based inks, which could be applied in forensic scenarios.
